# Supplementary material for: Splicing of enhancer-associated lincRNAs contributes to enhancer activity
Source: Life Sci Alliance. 2020 Feb 21;3(4):e202000663. doi: 10.26508/lsa.202000663 (PMC7035876; doi:10.26508/lsa.202000663)
Supplement: Supplementary file 2 [file LSA-2020-00663_Supplemental_Data_1.docx]

**SUPPLEMENTARY NOTE**

Given the relatively small number of stringently annotated elincRNAs, we investigated if the observed association between elincRNA splicing and enhancer activity could be recapitulated using a larger set of elincRNAs that we annotated using a more permissive criterion. Specifically, we considered a mESC lincRNA to be transcribed from an intergenic enhancer if its 5’end is within 500 bp of the enhancer. Using this approach, we identified 1983 elincRNAs of which 211 are multi-exonic (Supplementary Note Figure 1). Similarly, non-enhancer associated mESC-expressed lincRNAs (oth-lincRNAs, n=891) and protein-coding genes (n=14329) were defined as transcripts whose annotated 5'end is within 500 bp of a promoter (Supplementary Note Figure 1). We compared the chromatin signatures at enhancers associated with these transcripts to all remaining mESC enhancers. In general, we found enhancers that give rise to multi-exonic elincRNAs are associated with enriched enhancer chromatin signatures (Supplementary Note Figure 2), stronger impact on target expression (Supplementary Note Figure 2), preferential location at TAD boundaries and increased local chromosomal interaction (Supplementary Note Figure 3). Multi-exonic elincRNAs are also efficiently processed (Supplementary Note Figure 4), and splicing of 5'end exons is associated with increased transcription (Supplementary Note Figure 5), consistent with our previous analysis of the stringently defined set of elincRNAs.

Compared to the stringently defined set of elincRNAs presented in the main text, we found an overall reduction in the signal supporting the association between elincRNA splicing to their cognate enhancer activity. We attribute this reduction, that does not impact the conclusions, to the fact that elincRNAs in the extensive set were defined based on their proximity to active enhancers with a lack of well-defined and experimentally determined transcriptional start site (i.e using CAGE reads). This likely explains why elincRNA splicing and DNA-DNA contact density are still correlated but this correlation is no longer significant using the extensive set of elincRNAs (Figure 3B, D, E and Supplementary Note Figure 3B, D, E).


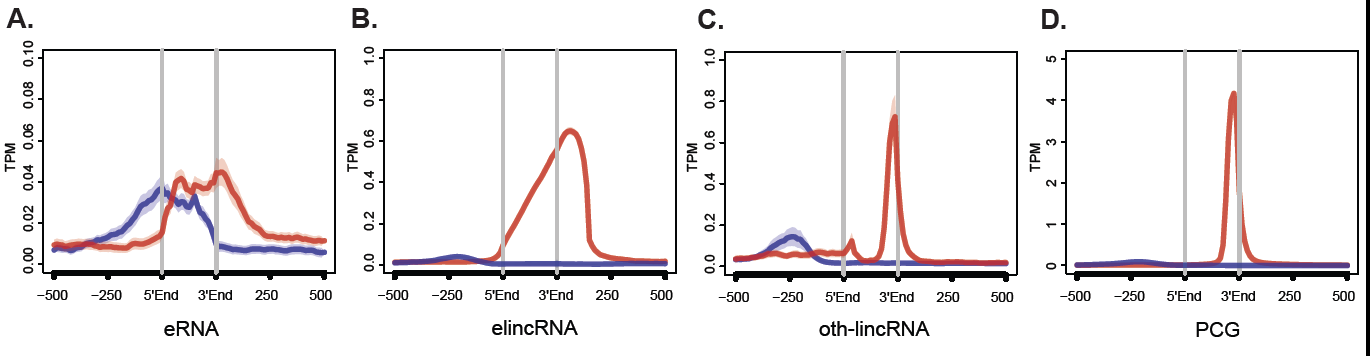


**Supplementary Note Figure 1. Extensive annotations of elincRNAs.** Metagene plots of CAGE reads centered at transcription initiation regions (TIRs) of (A) eRNAs, and promoters (estimated as -500bp to annotated gene TSS) of (B) elincRNAs, (C) other mESC-expressed lincRNAs (oth-lincRNAs) and (D) protein-coding genes (PCGs). Sense (red) and antisense (blue) reads denote those that map to the same or opposite strand, respectively, as the direction of their cognate TIRs/promoters.


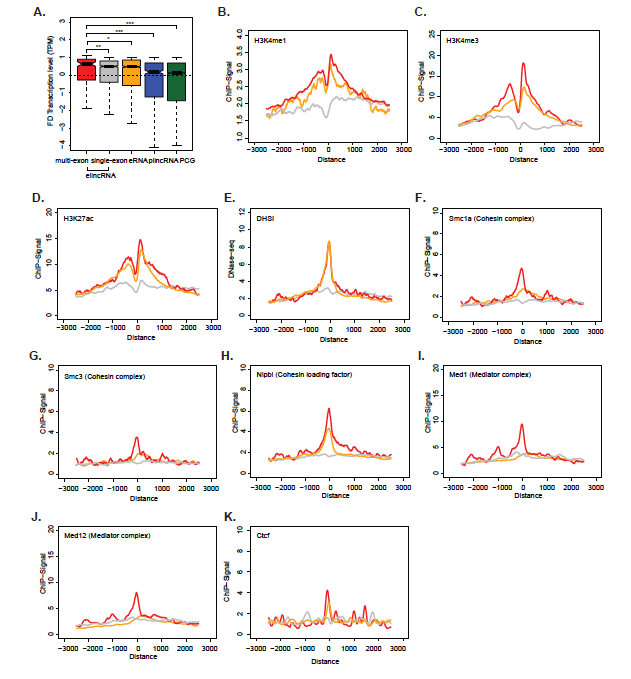


**Supplementary Note Figure 2. Multi-exonic elincRNAs are associated with higher enhancer activity.** (A) Distribution of the fold difference (FD) in transcription (measured as CAGE TPM) of the most proximal gene to multi-exonic (red) and single-exonic (grey) elincRNAs, eRNAs (yellow), other mESC-expressed lincRNAs (oth-lincRNAs, blue) and protein-coding genes (PCGs, green) both expressed in a same stage of embryonic neurogenesis. Fold difference of neighboring genes is calculated between the two cellular stages across neuronal differentiation, where the expression level of their reference locus (elincRNA, oth-lincRNA, or PCG) is maximal and minimal. Metagene plots of (B) H3K4me1, (C) H3K4me3, (D) H3K27ac, (E) DNase I hypersensitive sites (DHSI), (F) Smc1a, (G) Smc3, (H) Nipbl, (I) Med1, (J) Med12 and (K) Ctcf at transcription initiation regions of multi-exonic (red) and single-exonic (grey) elincRNAs, and eRNAs (yellow). Differences between groups were tested using a two-tailed Mann-Whitney *U* test. * *p* < 0.05; ** *p* < 0.01; *** *p* < 0.001; NS *p* > 0.05.

**
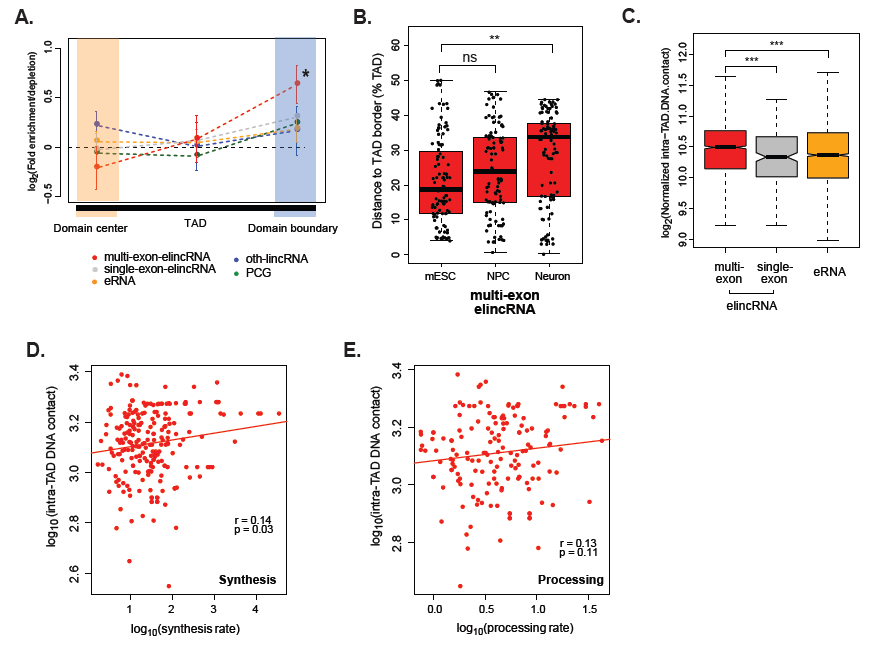
**

**Supplementary Note Figure 3. Multi-exonic elincRNAs are associated with modulation of local chromosomal architecture.** (A) Fold enrichment or depletion of multi-exonic (red) and single-exonic (grey) elincRNAs, eRNAs (yellow), other expressed lincRNAs (blue) and protein-coding genes (green) at boundaries (light blue shaded area) and center (light yellow shaded areas) of TADs. Significant fold differences are denoted with *(*p*<0.05, permutation test) and standard deviation is shown with error bars. (B) Distribution of the distance between multi-exonic elincRNA promoter (red) to the nearest TAD border in mESCs, neuronal precursor cells (NPCs) and neurons. (C) Distribution of the average amount of chromosomal contacts within mESC TADs that contain multi-exonic (red) and single-exonic (grey) elincRNAs and eRNAs (yellow). DNA-DNA contacts within multi-exonic elincRNA-containing mESC TADs (log10, Y axis) as a function of their respective (D) synthesis rate or (E) processing rate (log10, red points, Spearman’s correlation). Differences between groups were tested using a two-tailed Mann-Whitney *U* test. * *p* < 0.05; ** *p* < 0.01; *** *p* < 0.001; NS *p* > 0.05.

**
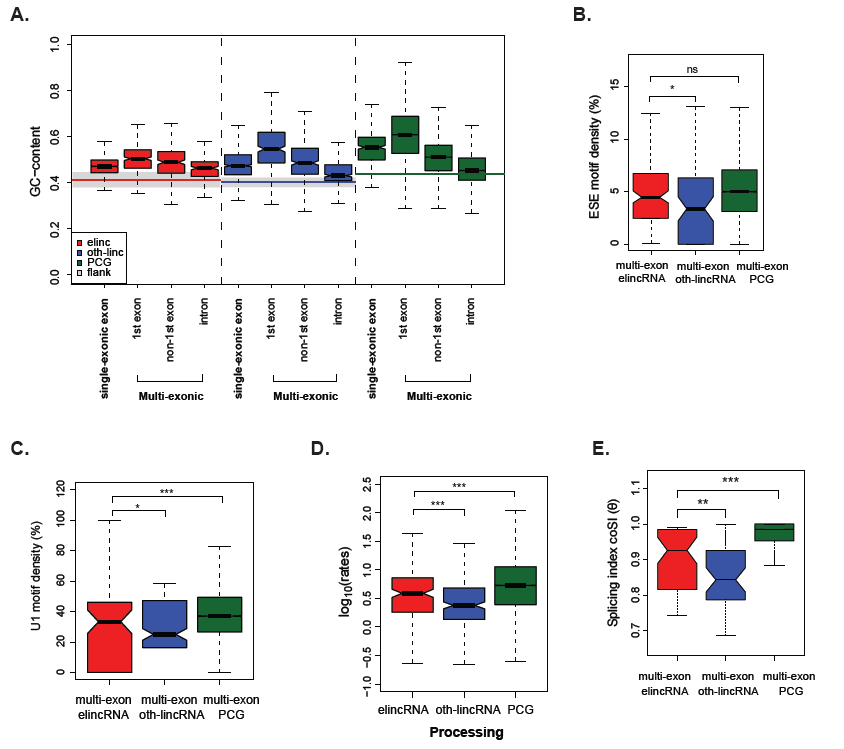
Supplementary Note Figure 4. elincRNA splicing is efficient.** (A) Distribution of the GC-content of exons and introns of single- and multi-exonic elincRNAs (red), other expressed lincRNAs (blue), protein-coding genes (green) and their respective flanking regions (grey). Distribution of the density of predicted (B) exonic splicing enhancers (ESEs) and (C) U1 spliceosome RNAs (snRNPs) within multi-exonic elincRNAs (red), other expressed lincRNAs (blue) and protein-coding genes (green). (D) Distribution of the average processing rates for elincRNAs (red), other expressed lincRNAs (blue) and protein-coding genes (green). (E) Distribution of the splicing index, coSI (θ) for multi-exonic elincRNAs (red), other expressed lincRNAs (blue) and protein-coding genes (green). Differences between groups were tested using a two-tailed Mann-Whitney *U* test. * *p* < 0.05; ** *p* < 0.01; *** *p* < 0.001; NS *p* > 0.05.

**
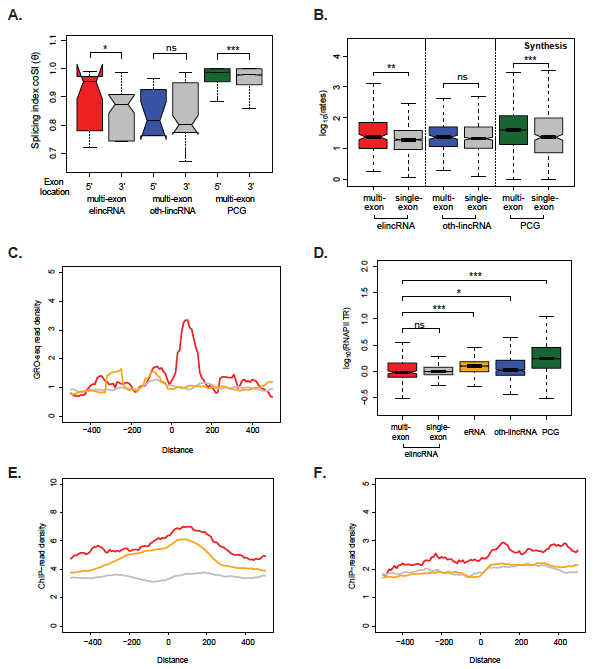
Supplementary Note Figure 5. elincRNA 5’end exon splicing associates with increased transcription.** (A) Distribution of the splicing index, coSI (θ) of introns located at the 5' or 3' ends of multi-exonic elincRNAs (red), other expressed lincRNAs (blue) and protein-coding genes (green). (B) Distribution of the RNA synthesis rates of multi-exonic elincRNAs (red), other expressed lincRNAs (blue) and protein-coding genes (green), as well as their single-exonic counterparts (grey). (C) Metagene plot of mESCs GRO-seq reads centered at transcription initiation regions (TIRs) of multi-exonic (red) and single-exonic (grey) elincRNAs and eRNAs (yellow). (D) Distribution of RNAPII travelling ratio (TR) for multi-exonic (red) and single-exonic (grey) elincRNAs, eRNAs (yellow), other expressed lincRNAs (blue) and protein-coding genes (green). Metagene plots and distribution (figure insets) of ChIP-seq reads for RNAPII with (E) phosphorylated-Serine 5 (S5P) and (F) phosphorylated Serine 2 (S2P) at their C-terminal domain centered at TIRs of multi-exonic (red) and single-exonic (grey) elincRNAs and eRNAs (yellow). Differences between groups were tested using a two-tailed Mann-Whitney *U* test. * *p* < 0.05; ** *p* < 0.01; *** *p* < 0.001; NS *p* > 0.05.
